# Ni‐Doped CuO Nanoarrays Activate Urea Adsorption and Stabilizes Reaction Intermediates to Achieve High‐Performance Urea Oxidation Catalysts

**DOI:** 10.1002/advs.202204800

**Published:** 2022-10-20

**Authors:** Hainan Sun, Jiapeng Liu, Hyunseung Kim, Sanzhao Song, Liangshuang Fei, Zhiwei Hu, Hong‐Ji Lin, Chien‐Te Chen, Francesco Ciucci, WooChul Jung

**Affiliations:** ^1^ Department of Materials Science and Engineering Korea Advanced Institute of Science and Technology (KAIST) Daejeon 34141 Republic of Korea; ^2^ Department of Mechanical and Aerospace Engineering The Hong Kong University of Science and Technology Kowloon Hong Kong 999077 China; ^3^ Wenzhou Institute University of Chinese Academy of Sciences Wenzhou Zhejiang 325001 China; ^4^ State Key Laboratory of Materials‐Oriented Chemical Engineering College of Chemical Engineering Nanjing Tech University Nanjing 211816 China; ^5^ Affiliation Max Planck Institute for Chemical Physics of Solids Nöthnitzer Strasse 40 01187 Dresden Germany; ^6^ National Synchrotron Radiation Research Center Hsinchu 30076 Taiwan; ^7^ Department of Chemical and Biological Engineering The Hong Kong University of Science and Technology Kowloon Hong Kong 999077 China; ^8^ HKUST Shenzhen‐Hong Kong Collaborative Innovation Research Institute Shenzhen 518049 China; ^9^ HKUST Energy Institute The Hong Kong University of Science and Technology Hong Kong 999077 China

**Keywords:** cation exchange, nanoarray structure, oxygen evolution reaction, transition and noble metals, urea oxidation reaction

## Abstract

Urea oxidation reaction (UOR) with a low equilibrium potential offers a promising route to replace the oxygen evolution reaction for energy‐saving hydrogen generation. However, the overpotential of the UOR is still high due to the complicated 6e^−^ transfer process and adsorption/desorption of intermediate products. Herein, utilizing a cation exchange strategy, Ni‐doped CuO nanoarrays grown on 3D Cu foam are synthesized. Notably, Ni‐CuO NAs/CF requires a low potential of 1.366 V versus a reversible hydrogen electrode to drive a current density of 100 mA cm^−2^, outperforming various benchmark electrocatalysts and maintaining robust stability in alkaline media. Theoretical and experimental studies reveal that Ni as the driving force center can effectively enhance the urea adsorption and stabilize CO*/NH* intermediates toward the UOR. These findings suggest a new direction for constructing nanostructures and modulating electronic structures, ultimately developing promising Cu‐based electrode catalysts.

## Graphical abstract

Utilizing a cation exchange strategy, metal‐doped CuO nanoarrays grown on three‐dimensional Cu foam were synthesized. Notably, Ni‐doped CuO nanoarrays requires a low potential of 1.366 V to drive 100 mA cm^−2^. Theoretical and experimental studies reveal that Ni as the driving force center can effectively enhance the urea adsorption and stabilize CO*/NH* intermediates toward the urea oxidation reaction.

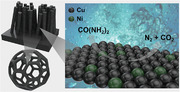

## 1 Introduction

Traditional water electrolysis is regarded as a promising technique for hydrogen production.^[^ ^1^ ^]^ However, the efficiency of electrochemical water splitting suffers from the anodic reaction (oxygen evolution reaction: OER) with a high overpotential due to the sluggish reaction kinetics (4OH^−^ → O_2_ + 2H_2_O + 4e^−^).^[^ ^2^ ^]^ Of note, it is difficult to make the free energy of the rate‐determining step become zero due to the unfavorable scaling relationships of OER intermediates.^[^ ^3^ ^]^ Even the state‐of‐the‐art OER electrocatalysts, such as precious metal‐ and NiFe‐based materials, require a minimum overpotential of ≈250–350 mV.^[^ ^3^ ^]^ To circumvent the shortcoming of high overpotentials toward the OER, replacing the OER with a more oxidizable anode reaction provides an avenue to generate hydrogen with less energy depletion.^[^ ^4^ ^]^ The urea oxidation reaction (UOR) has a relatively low thermodynamic equilibrium potential of 0.37 V, which is much lower than that of the OER (1.23 V).^[^ ^5^ ^]^ Therefore, a urea‐assisted hybrid water electrolysis technique is a good route to realize less energy consumption for hydrogen production and sewage treatment simultaneously.^[^ ^6^ ^]^

The kinetics of the UOR in alkaline media suffers from a six electron–proton coupled transfer step (CO(NH_2_)_2_ + 6OH^−^ → N_2_ + CO_2_ + 5H_2_O + 6e^−^).^[^ ^5a^ ^]^ The following are key factors to consider when designing high‐performance UOR electrocatalysts. First, the competitive reaction of the OER could largely impact the activity and selectivity of the UOR.^[^ ^7^ ^]^ Considering the reaction mechanisms, the adsorption abilities of the urea molecule and OH^−^ can reflect the onset potential of the UOR and OER, respectively.^[^ ^8^ ^]^ Moreover, the stabilization of key intermediates (CO* and NH*) is also as an important factor that significantly influences the UOR activity.^[^ ^9^ ^]^ Although precious metal catalysts can effectively enhance the activity, their high cost and scarcity limit large‐scale applications.^[^ ^9^, ^10^ ^]^ Moreover, in terms of water electrolysis in alkaline media, transition metal‐based materials have been demonstrated to have outstanding electrocatalytic activity and robust operational stability.^[^ ^11^ ^]^ Currently, high‐performance UOR electrocatalysts are still limited to Ni‐based materials, where Ni^3+^ (NiOOH) has been identified as the catalytically active site.^[^ ^5^, ^12^ ^]^ For example, Wang et al. found that introducing W into the Ni‐based catalyst can regulate the local charge distribution of Ni atoms, which contributes to the generation of high valence Ni^3+^ sites with high intrinsic activity.^[^ ^12a^ ^]^

Additionally, accelerating the charge/mass transfer‐ability and exposing sufficient surface active sites by constructing 3D nanostructured self‐supported electrodes is an effective approach that can also assure good electronic conductivity and rapid mass/charge transfer capabilities, and favors the high mechanical stability of catalysts for long‐term and cyclic usage.^[^ ^13^ ^]^ Cu belongs to first‐row transition elements with low toxicity. Cu foam is a good metal foam substrate to fabricate advanced self‐standing electrodes.^[^ ^4c^ ^]^ More importantly, compared with commercial Ni foam and Fe foam, in situ growth of a 1D nanostructure on the Cu foam surface by chemical oxidation or electrochemical oxidation reactions is appealing to construct active and durable electrodes.^[^ ^14^ ^]^ In a previous study, we found that among metallic Cu, Cu oxyhydroxide (Cu(OH)_2_), and Cu‐based oxides (Cu_2_O and CuO), CuO exhibits good activity, high selectivity, and robust stability toward the UOR in alkaline solutions.^[^ ^15^ ^]^ Therefore, constructing CuO nanowires as the UOR electrocatalysts has shown their inherent advantages. However, there is still much room for activity improvement to make CuO‐based materials that can serve as high‐performance UOR electrocatalysts. Moreover, the reports of a CuO‐based single phase with satisfactory UOR performance are limited.

In this work, metal‐doped CuO nanowire arrays were synthesized on a 3D commercial Cu foam (marked as M‐CuO NAs/CF) using a cation exchange strategy. The OER activities of CuO NAs/CF are improved upon the introduction of metal dopants, including noble and transition metals, to the host CuO. In contrast, interestingly, Ni dopant shows significantly superior UOR activity compared to other noble and transition metals. As a result, Ni‐CuO NAs/CF exhibits a low potential of 1.366 V versus reversible hydrogen electrode (RHE) at a current density of 100 mA cm^−2^ toward the UOR. Meanwhile, the electrode exhibits good operational stability with negligible performance degradation by maintaining a high current density of 100 mA cm^−2^. Experimental and theoretical calculations reveal that introducing Ni into the lattice of CuO can effectively activate urea adsorption and stabilize CO*/NH* intermediates rather than directly serving as active sites.

## 2 Results and Discussion

**Figure 1:**
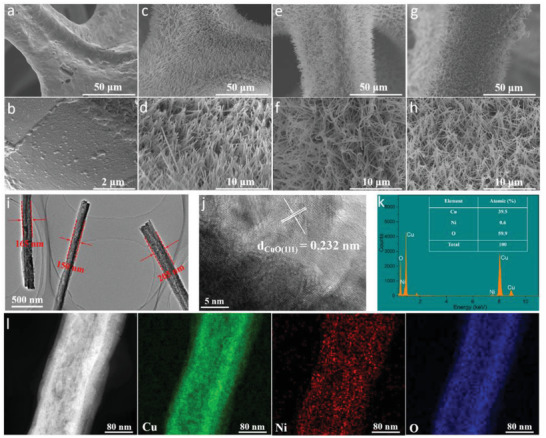
Structural characterizations. a,b) bare CF substrate, c,d) Cu(OH)_2_ NAs/CF, e,f) CuO NAs/CF, g,h) Ni‐CuO NAs/CF. i) TEM, and j) HRTEM image of Ni‐CuO NAs/CF and corresponding lattice spacing. k) EDX results. l) HRTEM and corresponding elemental mapping images for Ni‐CuO NAs/CF.

A cation exchange reaction coupled with calcination is as an effective strategy to synthesize metal‐doped nanostructures.^[^ ^9^, ^16^ ^]^ First, the in situ formation of Cu(OH)_2_ nanoarray structure was realized by the chemical oxidation of commercial Cu foam. Cu(OH)_2_ NAs/CF was immersed into a nickel chloride solution, and subsequently calcined in air (Experimental section). The scanning electron microscopy (SEM) images show that commercial CF has a smooth 3D surface (**Figure** **1**a,b). The nanoarray structure (Cu(OH)_2_ NAs/CF) was vertically and homogeneously grown onto the skeleton of the CF without the assistance of any binder after the chemical oxidation reaction (Figure 1c,d). For comparison, CuO NAs/CF was prepared by directly annealing Cu(OH)_2_ NAs/CF in air (Figure 1e,f). After the cation exchange reaction and subsequent thermal treatment, the morphology of the nanoarray structure was well maintained on the Ni‐Cu(OH)_2_ NAs/CF (Figure S1, Supporting Information) and Ni‐CuO NAs/CF (Figure 1g,h), respectively. X‐ray diffraction (XRD) spectra were used to track the phase transformation of the materials during synthesis. For Cu(OH)_2_ NAs/CF, the characteristic peaks at 16.6°, 23.7°, 33.9°, 36.0°, 38.2°, 39.7° and 53.2° were attributed to Cu(OH)_2_, and the diffraction peaks at 43.2°, 50.4° and 74.1° can be assigned to the Cu foam substrate.^[^ ^17^ ^]^ The phase transition from Cu(OH)_2_ to CuO was confirmed by the newly formed peaks at 35.5° and 38.7^o^ (Figure S2, Supporting Information). After the cation exchange reaction and calcination treatment, no additional peaks were found, and only diffraction peaks of CuO and Cu were observed in Ni‐CuO NAs/CF, indicating that, relative to CuO NAs/CF, Ni ions replaced Cu ions. Of note, due to the low doping amount, there is no significant change of XRD patterns after doping process.

The transmission electron microscopy (TEM) image also confirms a 1D nanowire structure with an average diameter of 170 ± 20 nm (Figure 1i). The high‐resolution TEM (HRTEM) image shown in Figure 1j displays lattice fringes with an interplanar distance of 0.232 nm corresponding to the (111) plane of CuO. The energy‐dispersive X‐ray (EDX) scanning reveals that the Ni‐CuO NAs/CF electrode has a low content of Ni (≈0.6 wt% of the total elements, Figure 1k). The EDX elemental mapping image shows that Ni, Cu, and O were uniformly distributed in the entire nanoarray (Figure 1l). A high‐angle annular dark‐field scanning TEM (HAADF‐STEM) analysis was conducted to further reveal the state of Ni and a lattice fringe of 0.21 nm was observed, corresponding to the (111) planes of CuO (Figure S3, Supporting Information). These results confirm that Ni was substituted in the lattice of CuO NAs to form a solid solution after the cation exchange reaction, which agrees with available reports.^[^ ^15^, ^16^, ^18^ ^]^

**Figure 2:**
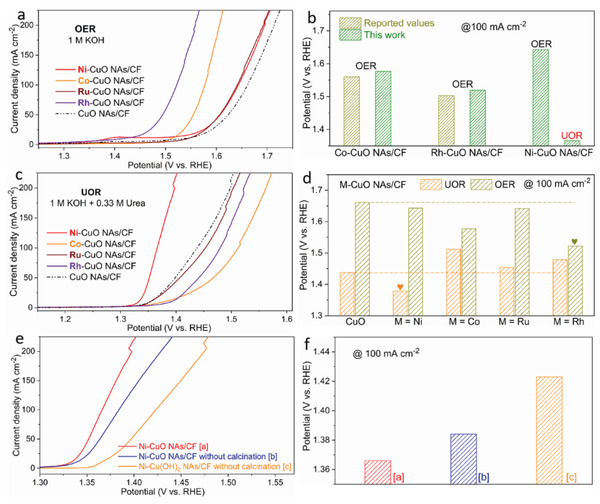
OER and UOR activities of study samples for comparison. a) OER curves of M‐CuO NAs/CF (M = Ni, Co, Ru, and Rh) and CuO NAs/CF. b) Comparison of potentials toward OER and UOR of electrodes, including reported values and values obtained in this work. c) UOR curves of the five electrodes. d) Comparison of the potential at 100 mA cm^−2^ toward OER and UOR. e) UOR curves of Ni‐CuO NAs/CF, Ni‐CuO NAs/CF without calcination, and Ni‐CuO NAs/CF without calcination. f) Comparison of potentials at 100 mA cm^−2^ of the electrodes in Figure 2e.

Element selection and subsequent heat treatment are key to achieving high performance. We further investigated the OER and UOR activities of M‐CuO NAs/CF (M = Co, Ni, Ru and Rh, Experimental Section), including transition and noble metal dopants (Figures S4 and S5, Supporting Information). Upon the introduction of an alien cation (dopant), M‐CuO NAs/CF (M = Ni, Co, Ru, and Rh) exhibited higher OER activity than that of the CuO NAs/CF electrode (**Figure** **2**a). The enhanced OER activity of multi‐metal oxides has been reported in previous literature.^[^ ^19^ ^]^ For example, the OER activity of the CuO nanoarray was greatly improved by Co (first‐row transition metal) doping.^[^ ^15b^ ^]^ Moreover, the precious metal Rh was demonstrated as an effective dopant to optimize the adsorption energies of the reactive intermediate toward the OER.^[^ ^16b^ ^]^ Of note, the OER activities of Co‐CuO NAs/CF and Rh‐CuO NAs/CF evaluated in our work match the reported data closely and the latter shows the best OER activity among the studied electrodes (Figure 2b).^[^ ^15^, ^16^ ^]^ In contrast, M‐CuO NAs/CF (M = Co, Ru, and Rh) exhibit decreased UOR activity compared with the pristine electrode (Figure 2c,d). Remarkably, Ni‐substitution exhibits a unique selectivity only in enhancing the UOR activity of CuO NAs/CF, highlighting the critical importance of element selection. Furthermore, the heat treatment is also demonstrated to be a key step. The CuO NAs/CF and Cu(OH)_2_ NAs/CF were directly immersed into a nickel chloride solution for 1 h and dried in a vacuum oven at 60 °C overnight, and they are denoted as Ni‐CuO NAs/CF without calcination and Ni‐Cu(OH)_2_ NAs/CF without calcination, respectively. As expected, the Ni‐CuO NAs/CF, which was annealed, exhibits the best UOR activity (Figure 2e). Specifically, the Ni‐CuO NAs/CF electrode requires 1.366 V to reach 100 mA cm^−2^, whereas Ni‐CuO NAs/CF without calcination and Ni‐Cu(OH)_2_ NAs/CF without calcination require high potentials of 1.384 and 1.423 V, respectively (Figure 2f).

**Figure 3:**
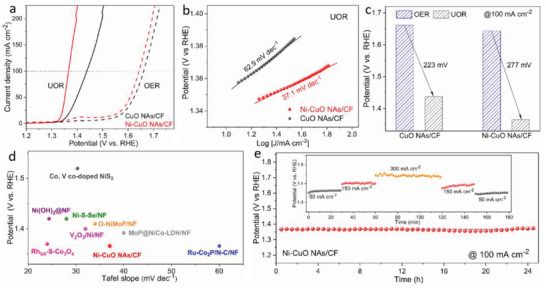
UOR performance. a) LSV curves of toward the OER and UOR in alkaline solutions. b) Tafel plots toward UOR. c) Potential values at 100 mA cm^−2^ toward the OER and UOR. d) Tafel slopes and potential at 100 mA cm^−2^ of Ni‐CuO NAs/CF and various electrocatalysts recently reported. e) Long‐term stability of Ni‐CuO NAs/CF under a fixed current density of 100 mA cm^−2^. Inset: multi‐step chronopotentiometric curves for Ni‐CuO NAs/CF.

To further investigate the effect of incorporated Ni, the electrocatalytic activities toward the OER (electrolyte: 1.0 m KOH) and UOR (electrolyte: 1.0 m KOH + 0.33 m urea) were evaluated. Notably, 0.33 m urea was selected to ensure the abundant urea concentration to obtain the maximal current density (Figure S6, Supporting Information).^[^ ^5a^ ^]^ **Figure** **3**a shows the linear sweep voltammetry (LSV) curves of as‐synthesized electrodes. In our previous work, we found that CuO has high reactivity and selectivity toward the UOR instead of the OER in alkaline media.^[^ ^15a^ ^]^ In particular, the potentials of the OER and UOR to reach a current density of 100 mA cm^−2^ on CuO NAs/CF were 1.661 and 1.438 V, respectively. The greatly decreased potential of 223 mV confirms the advantage of the UOR with reduced potential. When Ni was introduced, a low onset potential of 1.31 V was realized for Ni‐CuO NAs/CF toward the UOR, beyond which the current density increases sharply. Moreover, a potential of only 1.366 V is required to reach 100 mA cm^−2^ as well as a small Tafel slope of 37.1 mV dec^−1^, outperforming CuO NAs/CF (62.9 mV dec^−1^) (Figure 3b). Notably, compared with the OER activity, the potential for the UOR is only reduced by 18 mV to deliver 100 mA cm^−2^, suggesting that the Ni incorporation can effectively enhance the mass and intrinsic activity selectively toward the UOR (Figure 3c). The UOR activities of Ni‐CuO NAs/CF are one of the highest values among the recently reported UOR electrocatalysts (Figure 3d and Table S1, Supporting Information), placing the Ni‐CuO NAs/CF electrode among the most efficient catalysts toward the UOR in alkaline media. Of note, this work also presents one of the UOR electrocatalysts based on CuO, highlighting the great promising material functional materials beyond other transition metals.

The operational stability was also evaluated in order to confirm the potential practical application of the designed catalyst. Multi‐chronopotentiometry curves of Ni‐CuO NAs/CF collected at five fixed current steps (from 50 to 300 mA cm^−2^ and back to 50 mA cm^−2^) were used to investigate the mass transport during the UOR by observing the potential response. As displayed in Figure 3e, when the current density changes, the corresponding potential promptly answered to a constant value and remained unchanged for 30 min, revealing good mass transport abilities including fast diffusion of reagents and easy release of gas products. The time‐dependent potential curve tested at a constant current density of 100 mA cm^−2^ was used to evaluate the stability. The Ni‐CuO NAs/CF is capable of steadily driving a current density of 100 mA cm^−2^ at ≈1.36 V over 25 h. Cyclic voltammetry (CV) measurements were conducted to further demonstrate the operational stability of Ni‐CuO NAs/CF. The activity displayed negligible degradation after the 1000th CV cycle (Figure S7, Supporting Information). Together with the multi‐chronopotentiometry tests, the above results strongly confirm that the Ni‐CuO NAs/CF electrode endows superior electrocatalytic UOR stability. To highlight the improved UOR activity and energy‐saving H_2_ production feature of urea electrocatalysis, we assembled the two‐electrode system using the Ni‐CuO NAs/CF as the anode and Pt/C/NF as the cathode, respectively. As shown in Figure S8, Supporting Information, the full urea electrolysis system ensures a current density of 50 mA cm^−2^ at 1.41 V. By contrast, the alkaline water splitting requires a much higher potential of 1.60 V to obtain the same current density (Figure S8, Supporting Information). Furthermore, no significant activity degradation was observed after continuous operation at a current density of 50 mA cm^−2^ for 50 h, highlighting the superior stability of the anode electrode (Figure S9, Supporting Information).

**Figure 4:**
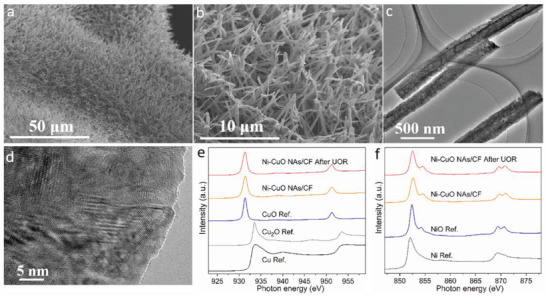
Characterizations after the UOR stability test. (a) and (b) SEM images, c) TEM images, d) HRTEM images of Ni‐CuO NAs/CF after the UOR testing. e) Cu *L*_2,3_ edge, and f) Ni *L*_2,3_ edge of Ni‐CuO NAs/CF before and after the UOR testing with different samples as references.

After the electrochemical reaction toward the UOR, the morphology and electronic structure of Ni‐CuO NAs/CF were examined. **Figure** **4**a,b shows SEM images of Ni‐CuO NAs/CF after the UOR stability test, revealing that the previous 3D structure is retained. The TEM image in Figure 4c reveals that the nanowire structure is well preserved. Moreover, the surface crystalline matrix is well presented without surface amorphization, indicating desirable operational stability (Figure 4d). To compare the surface electronic structure change before and after the UOR, soft X‐ray absorption spectra (sXAS) in total electron yield mode, which is a sensitive mode to detect surface information (≈5 nm depth), were used.^[^ ^20^ ^]^ As revealed in Figure 4e,f, the peak positions of Cu *L*_2,3_ and Ni *L*_2,3_ edges of Ni‐CuO NAs/CF after the UOR stability test were almost identical to those of the as‐prepared samples as well as the reference samples of Cu^2+^O and Ni^2+^O, indicating that there was not a significant composition change of Cu and Ni ions.^[^ ^21^ ^]^ Additionally, we also carried out the X‐ray photoelectron spectroscopy (XPS) analysis for Ni‐CuO NAs/CF before and after UOR (Figure S10, Supporting Information), which supports the results that Cu and Ni ions remained unchanged after the UOR.

**Figure 5:**
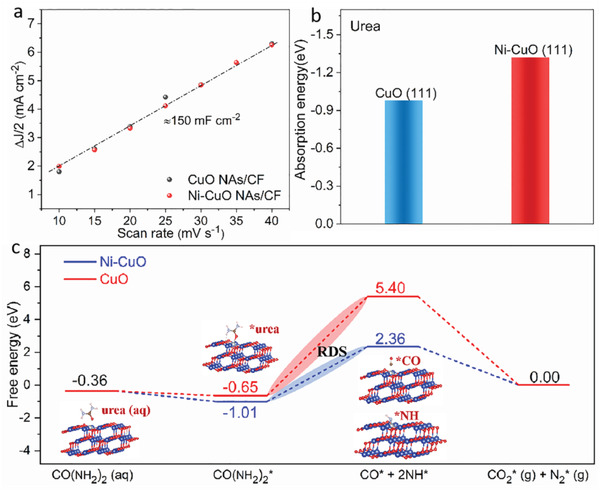
DFT calculations for the improved UOR activity. a) Plots showing the extraction of the double‐layer capacitance of CuO NAs/CF and Ni‐CuO NAs/CF. b) Adsorption energy of urea molecules on CuO (111) and Ni‐CuO (111). c) Reaction free energy profiles of UOR on CuO (111) and Ni‐CuO (111) surface. The inset shows the corresponding structural evolution of reaction intermediates adsorbed on Ni‐CuO (111). Color code: Cu (blue), Ni (gray), and O (red).

At present, considerable attention is being paid to developing Ni‐based materials as UOR electrocatalysts where high valence Ni (Ni^3+^) is traditionally identified as a catalytically active site. The value of the electrochemical double‐layer capacitance (*C*_dl_) can reflect the electrochemically active surface areas during the reaction.^[^ ^22^ ^]^ It is noteworthy that Ni‐CuO NAs/CF and CuO NAs/CF share similar values of *C*_dl_ (≈150 mF cm^−2^), suggesting that the Ni dopant does not contribute to more exposed active sites toward the UOR (**Figure** **5**a and Figure S11, Supporting Information). However, Ni‐CuO NAs/CF outperforms CuO NAs/CF in terms of electrochemical activity and reaction kinetics, demonstrating that the introduction of Ni can effectively improve the UOR intrinsic activity beyond its role as the active species. As demonstrated above, the adsorption abilities of the urea molecule reflect the onset potential of the UOR. Moreover, the stabilization of key intermediates (CO* and NH*) is demonstrated as an important factor greatly influencing the UOR activity.^[^ ^9^ ^]^ Therefore, to further understand the outstanding activity of Ni‐CuO NAs/CF toward the UOR under an alkaline environment, density functional theory (DFT) calculations were carried out to compare these two parameters between CuO and Ni‐CuO (Figure S12, Supporting Information). As displayed in Figure 5b, the adsorption energy of urea molecules on Ni‐CuO (111) is calculated to be −1.32 eV, which is much smaller than the value for CuO (111) (−0.98 eV). The much lower value demonstrates more favorable behavior of Ni‐CuO NAs/CF in terms of adsorbing urea than that of CuO NAs/CF, which enhances the intrinsic reactivity and improves the catalytic kinetics for the UOR. As reported, the adsorption of urea and the stabilization of the following intermediates, including CO* and NH*, have been identified as crucial parameters to determine the onset and overpotential of the UOR in alkaline solutions. The reaction free energy diagrams toward the UOR on CuO (111) and Ni‐CuO (111) surface are indicated in Figure 5c. It can be seen that the rate‐determining step (RDS) toward the UOR for both substrates is identified to be the dissociation of adsorbed CO(NH_2_)_2_* to CO* and NH* intermediates. In order to overcome this energy gap, CuO (111) requires a slight energy gain of 3.37 eV, a value much smaller than that on CuO (111). The decreased energy difference between CO(NH_2_)_2_* and CO* + NH* can be attributed to the stabilization of CO* and NH* intermediates along with high urea adsorption ability upon Ni introduction. This also verifies the remarkable UOR performance of Ni‐CuO NAs/CF. Importantly, the 1D structure of the Ni‐CuO NAs supported on Cu foam enables high catalytic activity and good stability. Therefore, constructing nanostructure and modulating the electronic structure jointly contribute to the high performance of the UOR. It was reported by Xu et al. that Rh modified CuO nanowire arrays on Cu foam exhibited unprecedented OER activity, which was attributed to the suitable binding ability of the OER intermediates, including O*, OH* and OOH*.^[^ ^16b^ ^]^ We further found that the UOR activity was decreased after introduction of Rh, indicating that electrocatalysts with a moderate adsorption energy of intermediates toward the OER may not be good candidates for the UOR due to the competitive relationship of the two reactions. The adsorption properties toward OER and UOR intermediates play a significant role in determining the electrocatalytic activities, highlighting the necessity to have an in‐depth understanding of these two reaction mechanisms to design advanced electrocatalysts.

## 3 Conclusions

In this work, we demonstrate that a cation exchange strategy coupled with calcination can be applied to synthesize metal‐doped CuO nanowire arrays grown on a 3D Cu foam. The element selection and subsequent heat treatment are key factors to achieve high‐performance toward the UOR in alkaline solutions. DFT calculations reveal that the UOR activity of CuO is greatly enhanced due to the enhanced urea adsorption property and stabilization of CO*/NH* intermediates toward the UOR by the Ni dopant. Remarkably, Ni‐CuO NAs/CF requires a low potential of 1.366 V to deliver 100 mA cm^−2^ while maintaining robust stability in alkaline media. This work has an in‐depth understanding of the OER/UOR competition relationship and highlights the promising CuO‐based materials as the next‐generation UOR electrocatalysts with a boosted activity and stability.

## 4 Experimental Section

### Synthesis of M‐CuO NAs/CF (M = Ni, Co, Ru, and Rh)

Cu(OH)_2_ NAs was first in situ grown on a Cu foam (CF) substrate by a chemical oxidation reaction, as reported previously.^[^ ^15^, ^16^, ^23^ ^]^ A piece of cleaned CF (1.0 × 1.0 cm^2^) was immersed in a solution of (NH_4_)_2_S_2_O_8_ (0.125 m) and NaOH (2.5 m) under room temperature for 30 min. After the chemical oxidation reaction, the blue electrode was washed by deionized water and ethanol and then dried overnight in a vacuum oven at 60 °C to obtain Cu(OH)_2_ NAs/CF. Next, M‐Cu(OH)_2_ NAs/CF (M = Ni, Co, Ru, and Rh) were prepared by dipping Cu(OH)_2_ NAs/CF into the solution of NiCl_2_, CoCl_2_, RuCl_3_, and RhCl_3_ (20 mL, 10 mm) for 1 h under room temperature. M‐Cu(OH)_2_ NAs/CF was then annealed in air at 200 °C for 1 h. CuO NAs/CF was prepared by directly annealing Cu(OH)_2_ NAs/CF in air at 200 °C for 1 h. The Pt/C on Ni foam (Pt/C/NF) electrode was prepared by the drop‐casting method.^[^ ^15a^ ^]^

### Physical Characterizations

XRD patterns were collected on a Bragg–Brentano diffractometer (Rigaku Ultima IV, Japan) using Cu K alpha radiation (*λ* = 1.506 Å). The surface morphology was detected using field emission SEM (Hitachi S‐4800). TEM measurements were conducted on a JEOL JEM‐2200FS operated at 200 kV. The high‐angle annular dark‐field scanning TEM (HAADF‐STEM) images were taken at the KAIST Analysis Center for Research Analysis (KARA) (Titan Cubed G2 60–300, FEI Company). Soft XAS was conducted at the beamline BL11A of the National Synchrotron Radiation Center (NSRRC) in Taiwan. X‐ray photoelectron spectroscopy (XPS) was acquired using a K‐alpha Thermo VG Scientific.

### Electrochemical Measurements

The electrochemical performance was measured in a three‐electrode system at an electrochemical workstation (Bio‐logic VMP‐300, France) with an Ag/AgCl and a carbon rod as reference and counter electrodes, respectively. The as‐prepared self‐supported electrodes directly served as the working electrode. Linear sweep voltammetry (LSV) was conducted at a scan rate of 2 mV s^−1^ and voltages were 95% iR corrected. CV tests were carried out between voltage of 0.15 and 0.25 V (versus Ag/AgCl electrode) with different scan rates (10, 15, 20, 25, 30, 35, and 40 mV s^−1^) to obtain *C*_dl_. The durability of Ni‐CuO NAs/CF was evaluated by CV tests (0.2‐0.8 V versus Ag/AgCl with a scan rate of 100 mV s^−1^) and chronoamperometry. The Ag/AgCl scale can be transformed into the RHE scale by the formula *E*_RHE_ = *E*_Ag/AgCl_ + 0.197 + 0.059 × pH.

### DFT Calculations

All the spin‐polarized first‐principle calculations were performed using the Vienna ab initio simulation package (VASP)^[^ ^24^ ^]^ with a plane‐wave basis set defined by a kinetic energy cutoff of 400 eV. The projector augmented wave (PAW)^[^ ^25^ ^]^ pseudopotentials with valence‐electron configurations of 1*s* ^1^, 2*s* ^2^2*p* ^2^, 2*s* ^2^2*p* ^3^, 2*s* ^2^2*p* ^4^ and 3*p* ^6^3*d* ^10^4*s* ^1^ were employed for H, C, N, O and Cu, respectively. The electron exchange‐correlation was described using the Perdew–Burk–Ernzerhof (PBE) functional^[^ ^26^ ^]^ under the generalized gradient approximation (GGA) scheme. The CuO substrate was modeled by a *p*(2 × 2) unit cell of CuO (111) containing three layers with an O—Cu—O configuration. As a result, there were three Cu atomic layers and six oxygen atomic layers, in which the bottom three layers were fixed to simulate the bulk properties and the uppermost six layers were fully relaxed. The CuO (111) surface was chosen because this surface has previously been reported to be thermodynamically stable with lower free energy.^[^ ^27^ ^]^ A vacuum space of 12 Å along the *c* direction was added for all substrates to avoid strong interactions between neighboring substrates. The Brillouin zone was sampled using a Gamma centered *k‐*mesh with spacing smaller than 2*π* × 0.04 Å^−1^ until the energy and force converged within 10^−5^ eV and 0.02 eV Å^−1^, respectively. To simulate the Ni‐CuO, one Cu atom in the top surface of CuO (111) was replaced with Ni. The adsorption energy was calculated as follows:

(1) $$E_{\mathrm{ads}}=E_{\text{slab-mol}}-E_{\mathrm{slab}}-E_{\mathrm{mol}}$$

where *E*_slab‐mol_ is the total energy of the adsorbate−substrate system; *E*_slab_ is the energy of the naked surface slab; and *E*_mol_ is the energy of an isolated molecule. Under this definition, a negative *E*_ads_ for a certain molecule implies that its adsorption is exothermic.

All free energies were calculated with respect to H_2_, N_2_, CO_2_, and H_2_O.^[^ ^28^ ^]^ For example, the free energy of urea was calculated using the following the reaction, where E represents the DFT energy, ZPE represents the zero‐point energy correction, S is the entropy effect due to vibration, and T is the standard temperature (298 K).

(2) $${\mathrm{CO}}_{2}\left(g\right)+N_{2}\left(g\right)+3H_{2}\left(g\right)\to \mathrm{CO}{\left({\mathrm{NH}}_{2}\right)}_{2}\left(g\right)+H_{2}O\left(l\right)$$

(3) $$\begin{array}{ccc} \Delta G_{\mathrm{CO}{\left({\mathrm{NH}}_{2}\right)}_{2}} & = & \left(E_{\mathrm{CO}{\left({\mathrm{NH}}_{2}\right)}_{2}}+E_{H_{2}O}-E_{{\mathrm{CO}}_{2}}-E_{N_{2}}-3\times E_{H_{2}}\right)\\ & & +\left(ZPE_{\mathrm{CO}{\left({\mathrm{NH}}_{2}\right)}_{2}}\;+\;ZPE_{H_{2}O}\;-\;ZPE_{{\mathrm{CO}}_{2}}\;-\;ZPE_{N_{2}}\;-\;3\;\times \;ZPE_{H_{2}}\right)\\ & & -T\times \left(S_{\mathrm{CO}{\left({\mathrm{NH}}_{2}\right)}_{2}}+S_{H_{2}O}-S_{{\mathrm{CO}}_{2}}-S_{N_{2}}-3\times S_{H_{2}}\right) \end{array}$$

## Conflict of Interest

The authors declare no conflict of interest.

## Supporting information

Supporting Information

## Data Availability

The data that support the findings of this study are available on request from the corresponding author. The data are not publicly available due to privacy or ethical restrictions.
