# Supplementary material for: Ni‐Doped CuO Nanoarrays Activate Urea Adsorption and Stabilizes Reaction Intermediates to Achieve High‐Performance Urea Oxidation Catalysts
Source: Adv Sci (Weinh). 2022 Oct 20;9(34):2204800. doi: 10.1002/advs.202204800 (PMC9731696; doi:10.1002/advs.202204800)
Supplement: Supplementary file 1 — Supporting Information [file ADVS-9-2204800-s001.pdf]

# Supporting Information

## Ni-doped CuO Nanoarrays Activate Urea Adsorption and Stabilizes Reaction Intermediates to Achieve High-performance Urea Oxidation Catalysts

Hainan Sun[1,#], Jiapeng Liu[2,#], Hyunseung Kim[1], Sanzhao Song[3], Liangshuang Fei[4], Zhiwei Hu[5], Hong-Ji Lin[6], Chien-Te Chen[6], Francesco Ciucci[2,7,8,9,*], WooChul Jung[1,*]

[1] Department of Materials Science and Engineering, Korea Advanced Institute of Science and Technology (KAIST), Daejeon, 34141, Republic of Korea

[2] Department of Mechanical and Aerospace Engineering, The Hong Kong University of Science and Technology, Kowloon, Hong Kong, China

[3] Wenzhou Institute, University of Chinese Academy of Sciences, Wenzhou, Zhejiang 325001, China

[4] State Key Laboratory of Materials-Oriented Chemical Engineering, College of Chemical Engineering, Nanjing Tech University, Nanjing, 211816, China

[5] Affiliation Max Planck Institute for Chemical Physics of Solids, Nöthnitzer Strasse 40, Dresden 01187, Germany

[6] National Synchrotron Radiation Research Center, Hsinchu 30076, Taiwan

[7] Department of Chemical and Biological Engineering, The Hong Kong University of Science and Technology, Kowloon, Hong Kong, China

[8] HKUST Shenzhen-Hong Kong Collaborative Innovation Research Institute, Shenzhen, China

[9] HKUST Energy Institute, The Hong Kong University of Science and Technology, Hong Kong SAR, China

*To whom correspondence should be addressed. E-mail: francesco.ciucci@ust.hk; wcjung@kaist.ac.kr

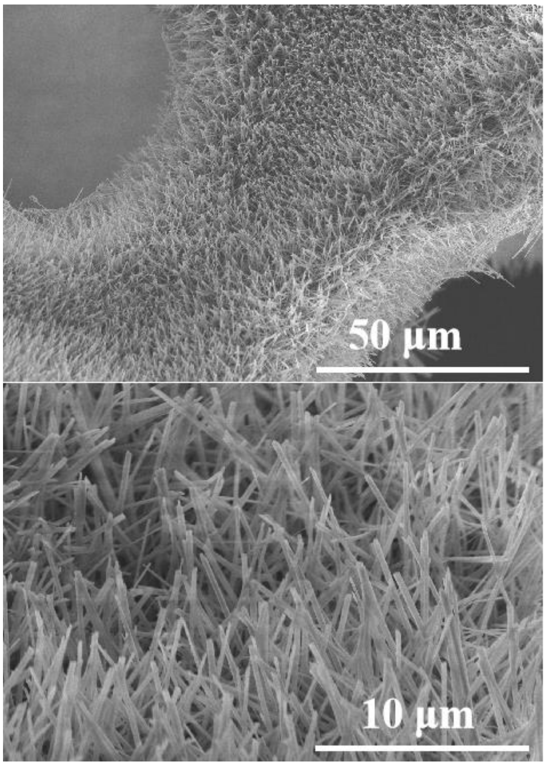


**Figure S1.** SEM images of Ni-$Cu(OH)_2$ NAs/CF.

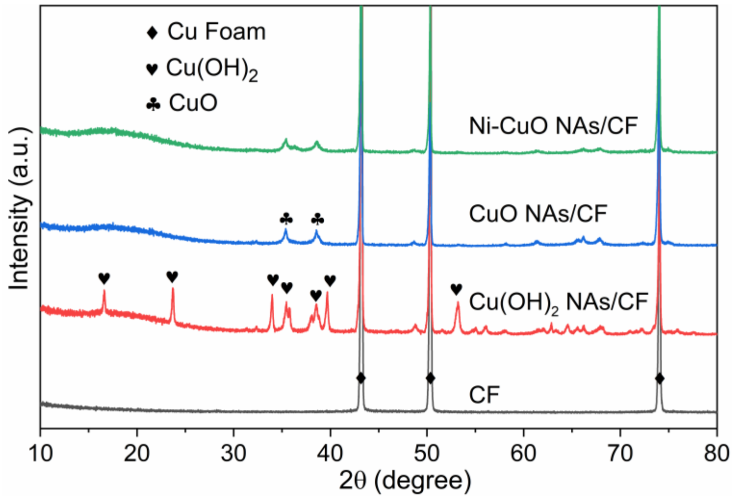


**Figure S2.** XRD patterns of bare CF, $Cu(OH)_2$ NAs/CF, CuO NAs/CF, and Ni-CuO NAs/CF.

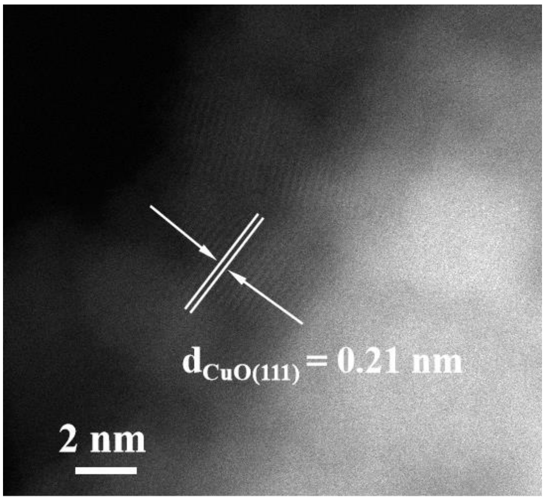


**Figure S3.** HAADF-STEM image of the Ni-CuO NAs/CF.

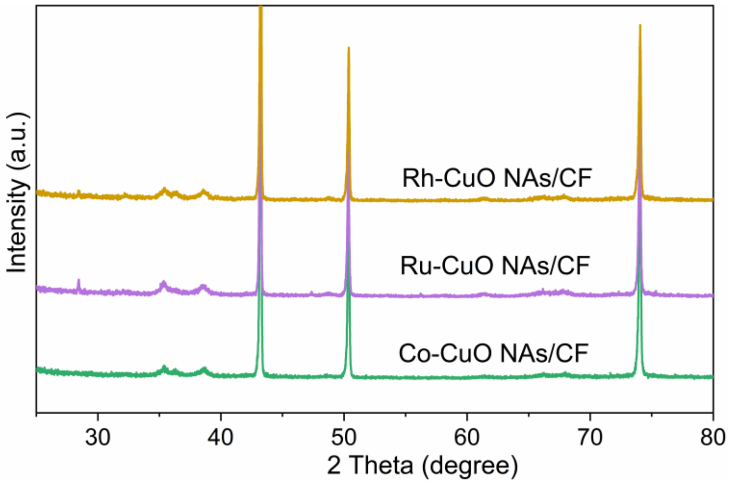


**Figure S4.** XRD patterns of Co-CuO NAs/CF, Ru-CuO NAs/CF, and Rh-CuO NAs/CF.

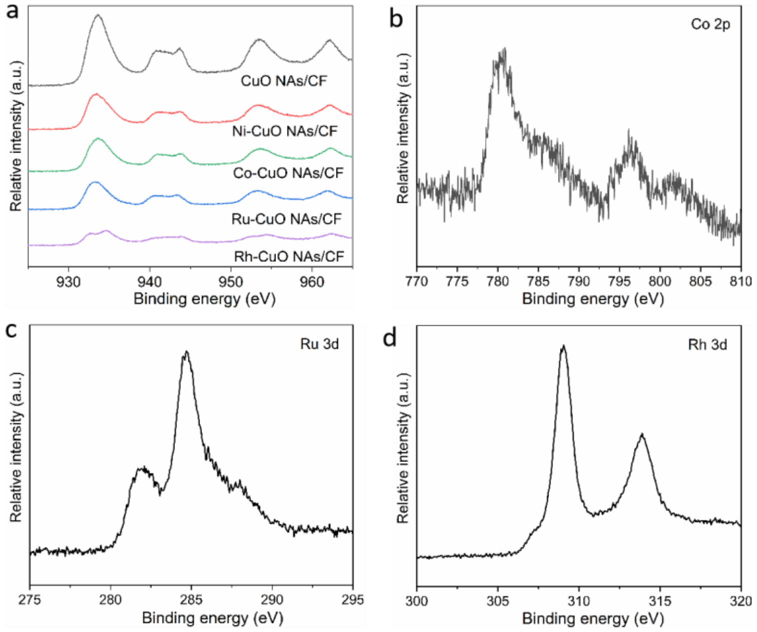


**Figure S5.** (a) Cu 2p XPS of CuO NAs/CF and M-CuO NAs/CF (M = Ni, Co, Ru, and Rh). (b) Co 2p XPS of Co-CuO NAs/CF. (c) Ru 3d XPS of Ru-CuO NAs/CF. (d) Rh 3d XPS of Rh-CuO NAs/CF.

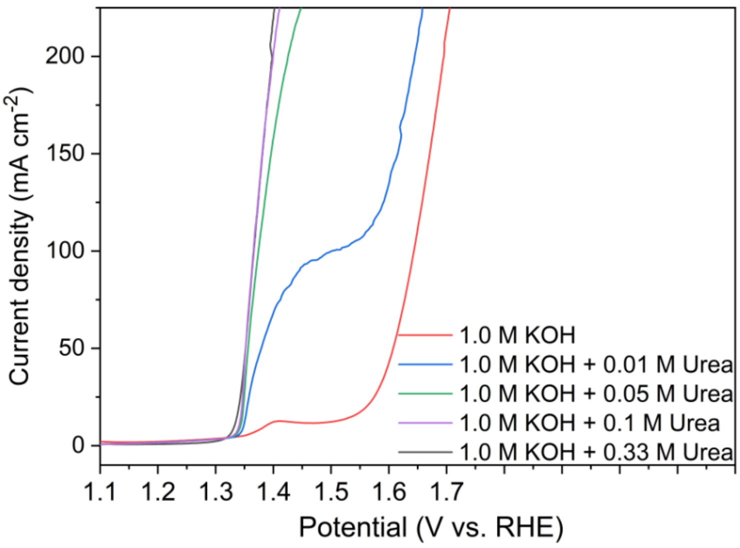


**Figure S6.** LSV curves of with different urea concentrations in 1.0 M KOH electrolyte.

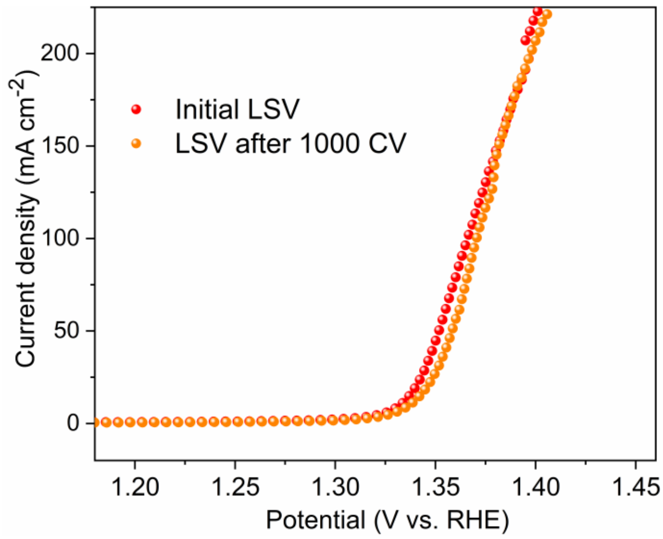


**Figure S7.** LSV curves collected before and after 1000th CV cycles for Ni-CuO NAs/CF.

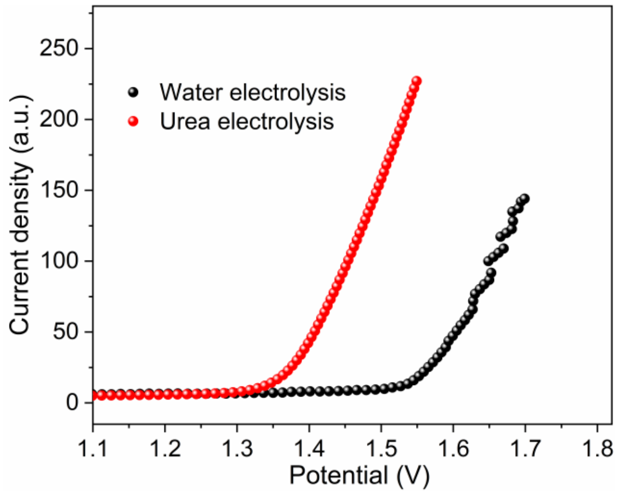


**Figure S8.** LSV curves for the Ni-CuO NAs/CF (anode)//Pt/C/NF (cathode) couple in 1.0 M KOH with and without 0.5 M urea.

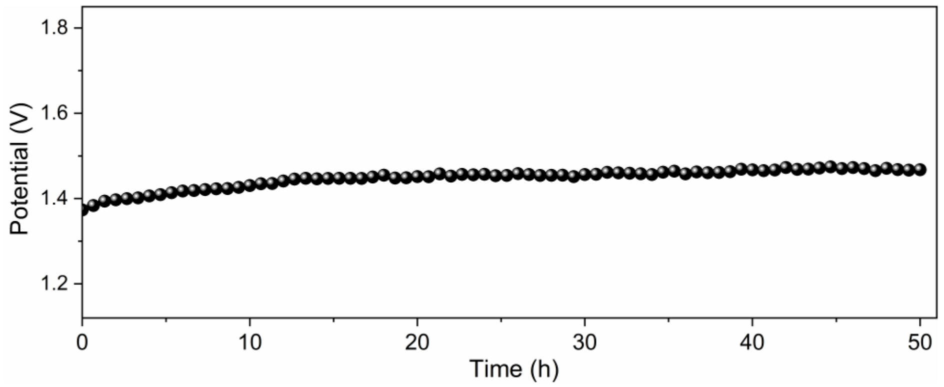


**Figure S9.** Chronopotentiometry curves of Ni-CuO NAs/CF//Pt/C/NF coupled with a fixed current density of 50 mA $cm^{-2}$ tested in a two-electrode configuration for 50 h.

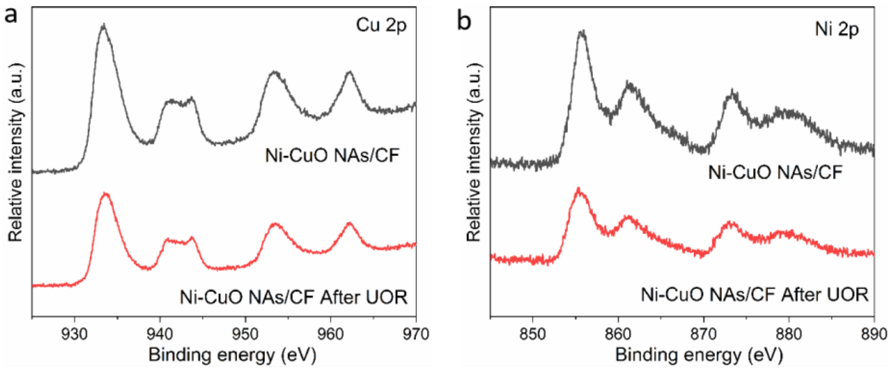


**Figure S10.** (a) Cu 2p and (b) Ni 2p XPS spectra of the Ni-CuO NAs/CF electrode before and after the UOR process.

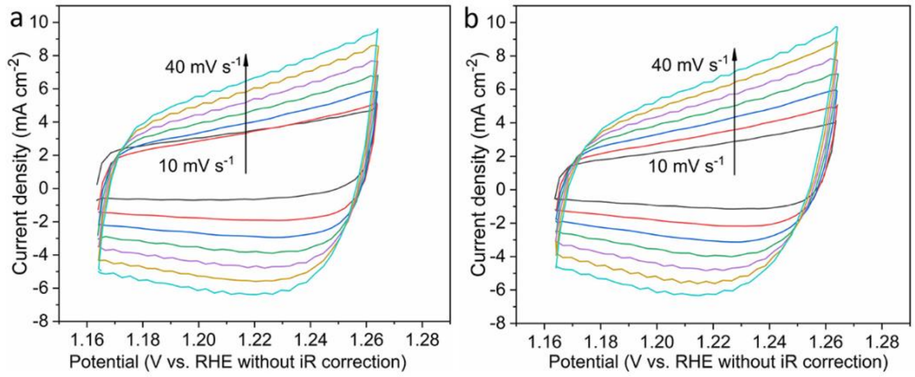


**Figure S11.** CV curves of (a) CuO NAs/CF and (b) Ni-CuO NAs/CF electrodes at different scan rates toward UOR.

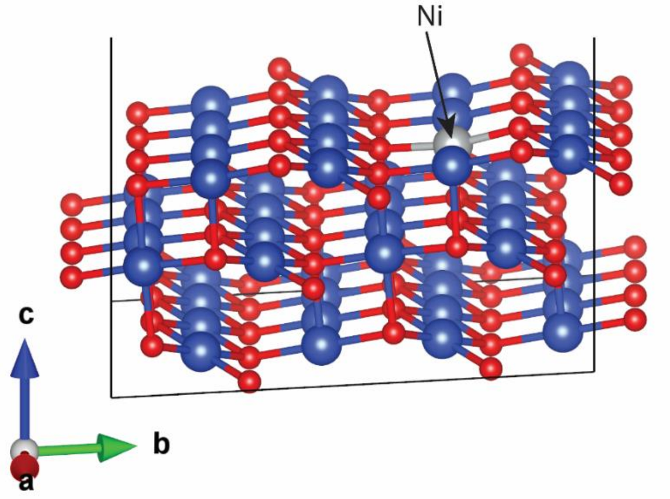


**Figure S12.** Structure models of (a) CuO and (b) Ni-CuO. Color code: Cu (blue), Ni (gray), and O (red). With the optimized CuO (111) slab in our previous work[1], we construct the Ni-CuO by replacing one surface Cu with Ni.

**Table S1.** Comparisons of the UOR performance of Ni-CuO NAs/CF electrode in terms of Tafel slope and potential at a current density of 100 mA $cm^{-2}$ with other reported transition-metal-based OER/UOR electrocatalysts.

| Catalysts | Potential at 100 mA $cm^{-2}$ (V) | Tafel slope (mV $dec^{-1}$) | Electrolyte | Ref. |
|---|---|---|---|---|
| Ni-CuO NAs/CF | 1.366 | 37.1 | 1.0 M KOH + 0.33 M Urea | This work |
| Co,V co-doped $NiS_2$ | 1.40 | 32 | 1.0 M KOH + 0.5 M Urea | [2] |
| $Ni(OH)_2$@NF | ≈1.42 | 24.4 | 1.0 M KOH + 0.3 M Urea | [3] |
| Ni-S-Se/NF | 1.42 | 28 | 1.0 M KOH + 0.5 M Urea | [4] |
| O-NiMoP/NF | ≈1.41 | 34 | 1.0 M KOH + 0.5 M Urea | [5] |
| $V_2O_3$/Ni/NF | 1.40 | 32 | 1.0 M KOH + 0.5 M Urea | [6] |
| MoP@NiCo-LDH/NF | 1.392 | 40 | 1.0 M KOH + 0.5 M Urea | [7] |
| $Rh_{SA}$-S-$Co_3O_4$ | ≈1.35 | 24 | 1.0 M KOH + 0.5 M Urea | [8] |
| Ru-$Co_2P$-C/NF | 1.366 | 60 | 1.0 M KOH + 0.5 M Urea | [9] |
| Rh SAC-CuO NAs/CF | ≈1.48 | 71.7 | 1.0 M KOH | [10] |
| Co-CuO NA/CF | 1.56 | 134 | 1.0 M KOH | [11] |
| Co-CuO | ≈1.51 | 118 | 1.0 M KOH | [12] |
| Cu@NiFe LDH | 1.511 | 27.8 | 1.0 M KOH | [13] |
| FeCoNi LDH/CuO/Cu | ≈1.49 | 63.8 | 1.0 M KOH | [14] |

| | | | | |
|---|---|---|---|---|
| CuO@CoOOH/CF | ≈1.64 | 51.7 | 1.0 M KOH | [15] |
| Ni@JXUN-4-NA/CF | ≈1.62 | 157 | 1.0 M KOH | [16] |

**References:**


[1] H. Sun, J. Liu, G. Chen, H. Kim, S. Kim, Z. Hu, J.-M. Chen, S.-C. Haw, F. Ciucci, W. Jung, *Small Methods* **2022**, *6*, 2101017.

[2] Z. Ji, Y. Song, S. Zhao, Y. Li, J. Liu, W. Hu, *ACS Catal.* **2022**, *12*, 569.

[3] L. Xia, Y. Liao, Y. Qing, H. Xu, Z. Gao, W. Li, Y. Wu, *ACS Appl. Energy Mater.* **2020**, *3*, 2996.

[4] N. Chen, Y.-X. Du, G. Zhang, W.-T. Lu, F.-F. Cao, *Nano Energy* **2021**, *81*, 105605.

[5] H. Jiang, M. Sun, S. Wu, B. Huang, C.-S. Lee, W. Zhang, *Adv. Funct. Mater.* **2021**, *31*, 2104951.

[6] Q. Zhang, B. Liu, L. Li, Y. Ji, C. Wang, L. Zhang, Z. Su, *Small* **2021**, *17*, 2005769.

[7] T. Wang, H. Wu, C. Feng, L. Zhang, J. Zhang, *J. Mater. Chem. A* **2020**, *8*, 18106.

[8] A. Kumar, X. Liu, J. Lee, B. Debnath, A. R. Jadhav, X. Shao, V. Q. Bui, Y. Hwang, Y. Liu, M. G. Kim, H. Lee, *Energy Environ. Sci.* **2021**, *14*, 6494.

[9] Y. Xu, T. Ren, K. Ren, S. Yu, M. Liu, Z. Wang, X. Li, L. Wang, H. Wang, *Chem. Eng. J.* **2021**, *408*, 127308.

[10] H. Xu, T. Liu, S. Bai, L. Li, Y. Zhu, J. Wang, S. Yang, Y. Li, Q. Shao, X. Huang, *Nano Lett.* **2020**, *20*, 5482.

[11] X. Xiong, C. You, Z. Liu, A. M. Asiri, X. Sun, *ACS Sustain. Chem. Eng.* **2018**, *6*, 2883.

[12] A. K. Mishra, D. Pradhan, *ACS Appl. Energy Mater.* **2021**, *4*, 9412.

[13] L. Yu, H. Zhou, J. Sun, F. Qin, F. Yu, J. Bao, Y. Yu, S. Chen, Z. Ren, *Energy Environ. Sci.* **2017**, *10*, 1820.

[14] Q. Ouyang, S. Cheng, C. Yang, Z. Lei, *J. Mater. Chem. A* **2022**, *10*, 11938.

[15] J. Hu, A. Al-Salihy, J. Wang, X. Li, Y. Fu, Z. Li, X. Han, B. Song, P. Xu, *Adv. Sci.* **2021**, *8*, 2103314.

[16] Z.-Q. Jiang, Y.-F. Li, X.-J. Zhu, J. Lu, T. Wen, L. Zhang, *Chem. Commun.* **2019**, *55*, 4023.
